# Hormonal Contraceptives and Migraine Frequency and Severity in Women: A Systematic Review and Meta-Analysis

**DOI:** 10.7759/cureus.107841

**Published:** 2026-04-27

**Authors:** Sama K Alju, Amirah Alqahtani, Nuha Alharbi, Rawan Munawir Farhan Alharbi, Farah Alsadoun, Meshari Mutlaq Ossman Alharbi, Abdullah Sameh Abdullah Almutairi, Ahlam Lafi Musaad Alharbi, Batool Hamoud M. Albishri, Khaled Ahmed Habab Alrshidi, Saleh Abdulaziz Kulaib Aljuhani, Nourah Alhamdan

**Affiliations:** 1 Department of Medicine, College of Medicine, Qassim University, Qassim, SAU; 2 Department of Internal Medicine, College of Medicine, Imam Mohammad Ibn Saud Islamic University (IMSIU), Riyadh, SAU

**Keywords:** estrogen withdrawal, hormonal contraceptives, hormone-free interval, menstrual migraine, systematic review and meta-analysis

## Abstract

Migraines affect a substantial proportion of female migraineurs in a menstrual pattern, yet the relationship between hormonal contraceptive use and migraine outcomes remains unclear. No prior systematic review has comprehensively synthesized quantitative data on migraine frequency and severity in hormonal contraceptive users. We systematically searched multiple electronic databases for studies reporting quantitative migraine outcomes in women using hormonal contraceptives. Random-effects meta-analyses using standardized mean differences examined within-subject comparisons (hormone-free interval versus hormone phase) separately from between-group comparisons (users versus non-users). Several studies of varying designs and sample sizes were included. The overall pooled estimate for migraine frequency showed no significant effect, with substantial heterogeneity across studies. Subgroup analysis revealed significant effect modification by comparison type. Within-subject studies demonstrated a marked increase in migraine frequency during the hormone-free interval, whereas between-group comparisons showed no significant difference between contraceptive users and non-users. Acute medication use was also significantly elevated during the hormone-free interval. These findings indicate that the hormone-free interval, rather than hormonal contraceptive use per se, represents a period of heightened migraine burden. Substantial heterogeneity and observational designs limit causal inference, and randomized trials comparing contraceptive regimens are needed to establish definitive conclusions.

## Introduction and background

Migraine is a disabling neurological disorder that ranks as the second leading cause of years lived with disability globally and the foremost cause among women of reproductive age [1]. The condition affects approximately 18% of women, with a two- to three-fold higher prevalence compared to men during the reproductive years [2, 3]. This pronounced sex difference emerges after menarche and persists throughout the reproductive lifespan, implicating female sex hormones as key modulators of migraine pathophysiology.

Among women with migraines, 18-25% experience attacks temporally associated with menstruation, a condition termed menstrual migraine [4, 5]. Perimenstrual attacks are clinically distinct from those occurring at other cycle phases, characterized by longer duration, greater severity, higher recurrence rates, and reduced responsiveness to acute treatment [6, 7].

The pathophysiology of menstrual migraine centers on the estrogen withdrawal hypothesis: the rapid decline in circulating estrogen preceding menstruation triggers migraine attacks through effects on the trigeminovascular system [8-10]. Sex hormone fluctuations also modulate migraine-related neuropeptides such as calcitonin gene-related peptide (CGRP) and pituitary adenylate cyclase-activating polypeptide-38 (PACAP-38), which may underpin hormone-dependent variation in migraine frequency and severity [11, 12].

In women using cyclic combined hormonal contraceptives, the standard 21/7 regimen introduces a predictable seven-day hormone-free interval during which exogenous estrogen is abruptly withdrawn. This pattern of hormone exposure creates a unique context for studying estrogen withdrawal migraine, as the timing and magnitude of hormone fluctuations are more predictable than in natural menstrual cycles [13]. The hormone-free interval in combined hormonal contraceptive users is characterized by both a more abrupt and a more pronounced estrogen decline compared with the natural cycle, potentially intensifying migraine vulnerability during this period [14]. These hormone-dependent mechanisms carry direct clinical relevance, as contraceptive regimen choice may modulate migraine burden in women with established migraine.

The relationship between hormonal contraceptive use and migraine outcomes remains incompletely understood. Some studies suggest that combined hormonal contraceptives may exacerbate migraine during the hormone-free interval [15], while others report minimal differences between contraceptive users and non-users [16, 17]. This apparent inconsistency may reflect methodological heterogeneity, including differences in study design, contraceptive formulations, and analytical approaches. Critically, the distinction between within-subject comparisons examining the same woman during different hormonal phases and between-group comparisons of different women based on contraceptive status may yield fundamentally different conclusions about hormonal effects on migraine.

To our knowledge, no prior systematic review has comprehensively synthesized quantitative data on migraine frequency and severity in women using hormonal contraceptives. Unlike prior reviews on menstrual migraine, this synthesis explicitly distinguishes within-subject comparisons (hormone-free interval vs hormone phase) from between-group comparisons (users vs non-users), addressing a key source of heterogeneity in the existing literature. This systematic review and meta-analysis aims to evaluate the impact of hormonal contraceptives on migraine frequency and severity in women with established migraine diagnoses, thereby informing contraceptive counseling and identifying priorities for future research.

## Review

Methods

Protocol

This systematic review was conducted in accordance with the Preferred Reporting Items for Systematic Reviews and Meta-Analyses (PRISMA) 2020 guidelines. A review protocol specifying eligibility criteria, search strategy, and analytical plan was developed a priori.

Eligibility Criteria

Studies were eligible for inclusion if they met the following criteria: (1) published in English from 2020 onwards, (2) enrolled women with an established migraine diagnosis at baseline (i.e., migraine present prior to or at the time of hormonal contraceptive exposure assessment), (3) assessed hormonal contraceptive use as an exposure or intervention, (4) reported quantitative migraine frequency (number of migraine days or attacks per defined time period) or severity (using validated scales) as an outcome, (5) included a comparison between hormonal contraceptive users and non-users, a before-after comparison within hormonal contraceptive users, or a comparison between different hormonal contraceptive regimens, (6) employed an observational or interventional study design, and (7) had full-text availability.

Studies were excluded if they: (1) focused exclusively on non-hormonal contraceptives (e.g., copper intrauterine device (IUD), barrier methods), (2) included only male participants, (3) did not report migraine-specific outcomes, (4) employed a review, editorial, letter, commentary, or abstract-only format, (5) addressed secondary headache disorders rather than primary migraine, (6) were published in languages other than English, (7) examined only migraine incidence, prevalence, or risk of developing migraine in general populations without reporting frequency or severity changes in women with established migraine, (8) lacked a comparator group or before-after design for assessing hormonal contraceptive effect, or (9) reported only categorical change in migraine frequency (e.g., "less frequent", "more frequent", "no change") without quantitative data. Studies relying solely on retrospective self-report without diary confirmation or validated questionnaire were eligible but categorized as low-quality evidence and subjected to sensitivity analysis. Operational definitions for migraine frequency, migraine severity, hormonal contraceptive exposure, and established migraine diagnosis are provided in the Appendix.

Search Strategy

A systematic search was conducted across three electronic databases: PubMed, Scopus, and Web of Science. The search covered the period from January 2020 to December 2025. Reference lists of included studies were screened to identify additional relevant articles. The search strategy combined terms related to three key concepts using Boolean operators (AND, OR). Hormonal contraceptive terms included: combined oral contraceptives, combined hormonal contraceptives, progestin-only pill, desogestrel, levonorgestrel, ethinyl estradiol, and hormone-free interval. Migraine terms included: migraine, menstrual migraine, menstrually related migraine, pure menstrual migraine, migraine with aura, migraine without aura, and catamenial migraine. Outcome terms included: frequency, severity, intensity, duration, migraine days, headache days, Migraine Disability Assessment (MIDAS), Headache Impact Test (HIT-6), and quality of life. The full search strategies for each database, including all Boolean operators, Medical Subject Headings (MeSH) terms, and filters, are provided in the Appendix. The final search was run on 26 December 2025.

Study Selection

Study selection was performed in two stages. In the first stage, titles and abstracts were screened independently by two reviewers to identify potentially eligible records. In the second stage, full-text articles of potentially eligible studies were retrieved and assessed against the predefined eligibility criteria. Discrepancies between reviewers were resolved through discussion or consultation with a third reviewer.

Data Extraction

Data were extracted independently by two reviewers using a standardized data extraction form. The following information was collected: (1) study characteristics, including first author, publication year, country, and study design; (2) population characteristics, including sample size, age, migraine subtype (with or without aura, menstrual or non-menstrual), and diagnostic criteria used; (3) exposure details, including type of hormonal contraceptive, dose, regimen (cyclic or continuous), duration of use, and comparator group; and (4) outcome data as specified below.

Outcome Measures

The primary outcomes were: (1) migraine frequency, defined as the number of migraine days or migraine attacks per month or per menstrual cycle, and (2) migraine severity, assessed using validated pain intensity scales such as the Visual Analog Scale (VAS), Numerical Rating Scale (NRS), or categorical scales (mild, moderate, severe).

Secondary outcomes included: (1) monthly headache days, encompassing both migraine and non-migraine headache; (2) attack duration measured in hours; (3) migraine-related disability assessed using the MIDAS questionnaire or the HIT-6; (4) quality of life measured using the Migraine-Specific Quality of Life Questionnaire (MSQ) or similar validated tools; (5) acute medication use, including number of days requiring triptans or analgesics; (6) presence and frequency of associated symptoms including aura, nausea, photophobia, phonophobia, and cutaneous allodynia; and (7) adverse events related to hormonal contraceptive use. Acceptable methods for outcome assessment included prospective headache diaries (paper or electronic), validated questionnaires, and clinical assessments.

Risk of Bias Assessment

Risk of bias was assessed using the Joanna Briggs Institute (JBI) Critical Appraisal Checklists appropriate to each study design: the 11-item checklist for cohort studies [7, 13, 17, 18], the eight-item checklist for analytical cross-sectional studies [12, 14], and the 10-item checklist for case-control studies [16]. Overall, the included studies demonstrated moderate methodological quality. None of the included studies were excluded based on quality assessment; however, methodological heterogeneity was considered when interpreting pooled estimates.

Data Synthesis

A qualitative synthesis was performed to summarize the characteristics and findings of included studies. Clinical and methodological heterogeneity were assessed to determine the feasibility of meta-analysis. Where sufficient homogeneity existed, a random-effects meta-analysis was planned using the restricted maximum likelihood (REML) estimator. Effect measures included odds ratios (OR) for dichotomous outcomes and mean differences (MD) or standardized mean differences (SMD) for continuous outcomes (using baseline standardization), with corresponding 95% confidence intervals. Details regarding imputation methods are provided in the Appendix.

Heterogeneity was quantified using the I² statistic and Cochran's Q test, with I² values of 25%, 50%, and 75% representing low, moderate, and high heterogeneity, respectively. Subgroup analyses were planned according to (1) comparison type (within-subject versus between-group) and (2) study design (prospective cohort versus cross-sectional versus case-control). Sensitivity analyses were planned to assess robustness by applying a leave-one-out approach. Publication bias was not assessed as the number of included studies was <10. The choice of effect measure, random-effects model with REML estimator, subgroup variables, sensitivity analysis, and publication bias threshold were specified a priori in the review protocol. All analyses were conducted using R v4.3 (R Foundation, Vienna, Austria) [19].

Results

Study Selection

**Figure 1:**
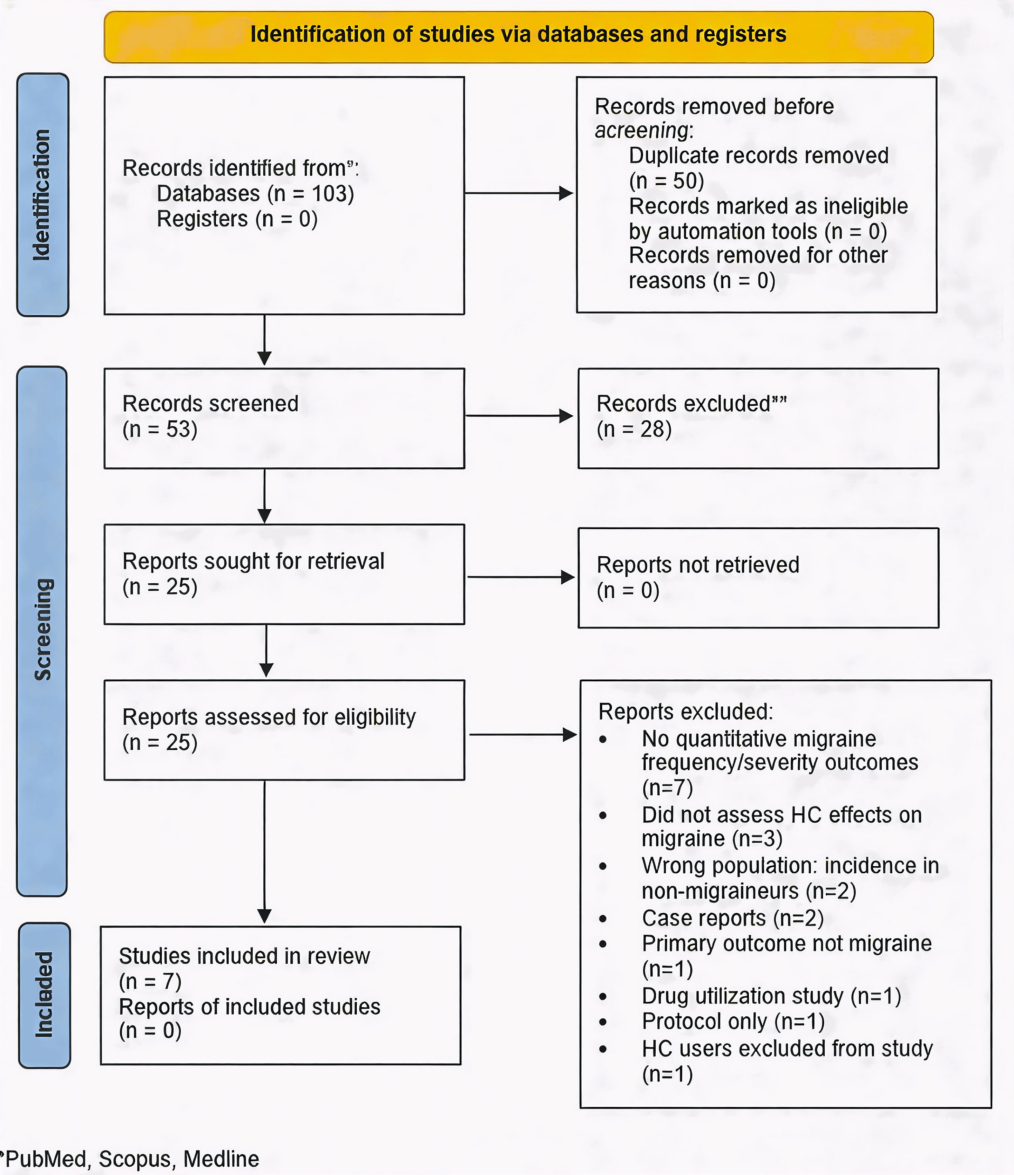
PRISMA flow chart for the included studies PRISMA - Preferred Reporting Items for Systematic Reviews and Meta-Analyses; HC - hormonal contraceptive

A total of 103 records were identified through database search (Figure 1). After exclusion of duplicates, 53 abstracts were screened for eligibility. Twenty-eight studies were further excluded: reviews or non-original research (n=17), non-hormonal contraceptive hormonal exposures (n=3), outcomes other than migraine frequency or severity (n=3), no assessment of hormonal contraceptive use (n=1), and other reasons including consensus statements, commentaries, wrong population, and drug utilization studies (n=4).

Study Characteristics

Seven studies met the inclusion criteria, comprising five prospective cohorts [7, 13, 14, 17, 18], one cross-sectional matched cohort [12], and one population-based case-control study [16]. Sample sizes ranged from 28 to 1,531 women with migraine, with mean ages spanning 25 to 40 years. All studies required International Classification of Headache Disorders 3 (ICHD-3) criteria for migraine diagnosis, though assessment methods varied from specialist interviews and prospective diaries to validated questionnaires. Hormonal contraceptive exposure was heterogeneous: four studies examined combined hormonal contraceptives in 21/7 regimens [7, 12, 13], one included both standard and extended combined oral contraceptive (COC) regimens [17], one grouped various contraceptive types [18], and one did not specify formulations [16]. Study designs included within-subject comparisons of hormone-free intervals versus hormone phases, between-group comparisons of HC users versus non-users, and comparisons based on timing of migraine onset relative to combined hormonal contraceptive (CHC) initiation (Table 1).

**Table 1:** Characteristics of included studies BTX-A - botulinum toxin A; CHC - combined hormonal contraceptive; CM - chronic migraine; COC - combined oral contraceptive; EE - ethinylestradiol; EM - episodic migraine; HC - hormonal contraceptive; HFI - hormone-free interval; ICHD-3 - International Classification of Headache Disorders 3rd edition; IQR - interquartile range; IUD - intrauterine device; MM - menstrual migraine; MRM - menstruation-related migraine; Mwa - migraine with aura; MwoA - migraine without aura; PMM - pure menstrual migraine.

Study	Design, country	N	Age, years	Migraine subtype	HC type & regimen	Comparison & follow-up
Merki-Feld et al., 2020 [13]	Prospective cohort, Switzerland	28	28 ± 5.3	MRM (n=25), PMM (n=3); ICHD-3 confirmed; 96.5% no/rare aura	CHC 21/7: oral (n=22), vaginal ring (n=6); EE 20–35 mcg	Within-subject: HFI vs. hormone phase; 3 cycles (84 days); daily diaries
Merki-Feld et al., 2022 [14]	Prospective cohort, Switzerland	48 (28 diaries)	26.5 ± 5.0	MRM (n=45), PMM (n=3); ICHD-3; 72.9% family history positive	CHC 21/7: oral/patch/ring; dose not specified	Within-subject + between-group (migraine before vs. after CHC start); 3 cycles
Storch et al., 2024 [12]	Cross-sectional matched cohort, Germany	60 migraine, 60 controls	26.5 (M-RMC), 25.0 (M-COC)	Episodic MRM per ICHD-3; 37–57% with aura; ≥3 migraine days/month	M-COC: COC 21/7, EE 0.03 mg; M-RMC: natural cycle (28±2 days)	Between-group: COC vs. natural cycle; 2 visits at perimenstrual and periovulatory phases
Chalmer et al., 2023 [16]	Population-based case-control, Denmark	1,531	38.7 ± 8.7	Pure MM (n=410), MRM (n=1,037); ICHD-3 via validated questionnaire	HC-related MM (n=298): type/dose not specified; Spontaneous MM (n=1,233): no HC	Between-group: HC-related vs. spontaneous MM; cross-sectional
van Casteren et al., 2021 [7]	Prospective cohort, Netherlands	500	40.5 ± 8.9	MwoA 53%, MwA 37%, CM 9%; MRM 56%; ICHD-3	COC with pill-free interval (n=65, 13%); natural cycle (n=435, 87%)	Between-group: HC vs. non-HC; 1–3 cycles; daily e-diary
Mas-de-les-Valls et al., 2025 [18]	Retrospective cohort with e-Diary, Spain	113 (89 with HC data)	39.0 (IQR 33–45)	EM 35%, CM 65%; 21% with aura; all on anti-CGRP/BTX-A	Contraceptive users (n=25): mixed types (pills/patch/ring/IUD); non-users (n=64)	Between-group: HC vs. non-HC; 3 months; validated e-Diary
Verhagen et al., 2022 [17]	Prospective cohort, Netherlands	607	39.7 ± 7.6 (non-HC), 33.1 ± 9.8 (HC)	37% with aura; ICHD-3 verified by headache specialist	COC (n=203): 21/7 (n=54) or extended (n=149); natural cycle (n=404)	Between-group: HC vs. non-HC; median 84 days; daily e-diary

Study-level migraine outcomes across the included studies are summarized in Table 2.

**Table 2:** Summary of migraine outcomes CHC - combined hormonal contraceptive; CI - confidence interval; HC - hormonal contraceptive; HFI - hormone-free interval; MM - menstrual migraine; MRM - menstruation-related migraine; OR - odds ratio; PACAP-38 - pituitary adenylate cyclase-activating polypeptide-38; VAS - Visual Analog Scale

Study	Migraine frequency	Migraine severity	Attack duration & medication use	Key findings
Merki-Feld et al., 2020 [13]	4.11 ± 2.81z days/cycle; HFI: 2.18 days/week vs. hormone phase: 0.64 days/week (p<0.0001). Relative risk peaked at 4.9-fold on HFI day 5 (95% CI 2.28–10.34).	Pain score 1.98 (HFI) vs. 1.78 (hormone phase) on 0–3 scale (p<0.03). 45% of severe attacks occurred during HFI.	Prolonged attacks (>24h): 0.29/week (HFI) vs. 0.057/week (hormone phase, p<0.0001); 75% occurred in HFI. Medication: 1.61 vs. 0.55 days/week (p<0.0001).	Migraine burden concentrated in HFI with a 4-fold increased risk on days 3–6. Recommend short-term prevention starting on day −2.
Merki-Feld et al., 2022 [14]	Group 1 (migraine before CHC): 5.0 ± 3.1 days/month vs. Group 2: 3.5 ± 2.1 (p=0.048). HFI vs. hormone phase: 2.1 vs. 0.7 days/week (p<0.001).	72.7% of Group 1 reported MRM more painful vs. 43.4% in Group 2 (p=0.015). Weekly pain score was higher in HFI (4.4 vs. 1.3, p<0.001).	Duration was similar between groups (25.2 vs. 29.8h, p=0.86). Good medication response: 31.8% (Group 1) vs. 65.2% (Group 2, p=0.05).	Women with migraine onset before CHC have higher frequency, more painful attacks, and poorer medication response. Consider progestin-only alternatives.
Storch et al., 2024 [12]	Median 4.0 days/month (M-RMC) vs. 5.8 (M-COC); not statistically compared.	Not reported.	Not reported.	PACAP-38 significantly higher in the natural cycle group vs. COC users during perimenstrual phase (2547 vs. 1504 pg/mL, p=0.040), suggesting COC modulates migraine biomarkers.
Chalmer et al., 2023 [16]	No difference: 60.1% vs. 60.9% had 1–3 days/month (OR 0.91, p=0.56); 13.8% vs. 14.3% had 4–7 days/month (OR 0.83, p=0.43).	VAS similar: 7.77 vs. 7.90 (OR 0.95, p=0.24). Pain characteristics (unilateral, pulsatile) comparable.	Duration similar: 63.8% vs. 54.9% lasted 4–24h. Triptan response: 79.4% vs. 82.9% (OR 0.97, p=0.93).	HC-related and spontaneous MM clinically similar except osmophobia less common in HC-related MM (30.4% vs. 43.3%, OR 0.58, p<0.001).
van Casteren et al., 2021 [7]	5.5 ± 4.2 migraine days/month overall. No direct HC vs. non-HC comparison reported.	Pain coping similar: non-HC 5.36 vs. HC 5.18 on 0–10 VAS.	Duration similar: 18.4h (non-HC) vs. 18.5h (HC). Analgesic use numerically higher in HC (50.1% vs. 41.4%). 2h pain-free: 30.0% vs. 25.6%.	Minimal differences between HC and non-HC groups; study not powered for this comparison. Perimenstrual attacks longer in HC users (60% vs. 31% >24h).
Mas-de-les-Valls et al., 2025 [18]	Daily headache probability: 61% (HC) vs. 50% (non-HC), OR 1.049, p=0.389. Daily migraine: 36% vs. 30%, OR 1.072, p=0.173.	Not reported by HC status.	Not reported by HC status.	HC users had significantly higher headache risk during ovulation (p=0.049) and perimenstrual windows (OR 1.64, p<0.001); non-users higher risk post-menstruation (p<0.001).
Verhagen et al., 2022 [17]	Median 5.0 days/month in both groups (non-HC vs. HC); no statistical comparison reported.	Not reported.	Duration trends similar in HC subgroup but not statistically significant.	Migraine frequency similar regardless of HC. Self-reported MM has poor accuracy (specificity 21%); a diary-based diagnosis essential.

Risk of Bias

Of the seven included studies, six were rated as high quality [7,12,14,16-18] and one as moderate quality [13]. Common strengths included the use of ICHD-3 diagnostic criteria, validated outcome measurements, and appropriate statistical analyses. The main limitations were unclear confounder adjustment and handling of missing data [13] and a lack of formal case-control matching [16]. For cohort studies examining within-subject changes in migraine characteristics, the item assessing whether participants were outcome-free at baseline was deemed not applicable, as these evaluated attack patterns in women with established migraine rather than incidence (Table 3).

**Table 3:** Risk of bias assessment results Y - yes (Low risk); N - No (High risk); U - Unclear; NA - Not Applicable: – - Item not in checklist for this design

Assessment criteria	Merki-Feld et al., 2020 [13]	van Casteren et al., 2021 [7]	Verhagen et al., 2022 [17]	Mas-de-les-Valls et al., 2025 [18]	Merki-Feld et al., 2022 [14]	Storch et al., 2024 [12]	Chalmer et al., 2023 [16]
Design	Cohort	Cohort	Cohort	Cohort	Cross-sectional	Cross-sectional	Case-control
1. Similar/comparable groups	Y	Y	Y	Y	Y	Y	Y
2. Exposure assignment	Y	Y	Y	Y	Y	Y	N
3. Exposure measurement valid	Y	Y	Y	Y	Y	Y	Y
4. Confounders identified	Y	Y	Y	Y	Y	Y	Y
5. Confounders addressed	U	Y	Y	Y	Y	Y	Y
6. Outcome-free at start	NA	NA	NA	NA	Y	Y	Y
7. Outcome measurement valid	Y	Y	Y	Y	Y	Y	Y
8. Follow-up duration/exposure period	Y	Y	Y	Y	Y	Y	Y
9. Follow-up complete	Y	Y	Y	Y	–	–	Y
10. Missing data handled	U	Y	Y	Y	–	–	Y
11. Statistical analysis appropriate	Y	Y	Y	Y	Y	Y	Y
Total	8/11	10/11	10/11	10/11	8/8	8/8	9/10
Quality rating	Moderate	High	High	High	High	High	High

Quantitative Synthesis

**Figure 2:**
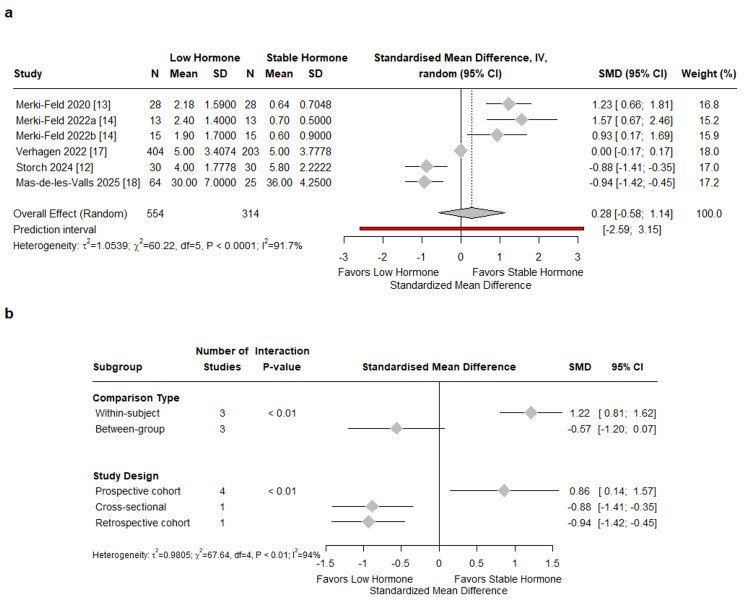
Meta-analysis of migraine frequency in women with menstrual migraine [12-14, 17, 18] (a) Forest plot comparing hormonal contraceptive (HC) users versus non-users, with within-subject studies comparing hormone-free interval (HFI) to hormone phase, and between-group studies comparing non-HC to HC users. Standardized mean differences (SMD) with 95% confidence intervals calculated using a random-effects model (inverse variance method, REML estimator); (b) Subgroup analyses stratified by comparison type and study design.

Migraine frequency: Six studies comprising 868 participants were included in the analysis of migraine frequency (Figure 2). The pooled standardized mean difference was 0.28 (95% CI: -0.58 to 1.14), with substantial heterogeneity across studies (I²=91.7%, τ²=1.05, χ²=60.22, df=5, p<0.0001).

Subgroup analysis by comparison type revealed significant effect modification (p<0.01). Within-subject studies comparing the hormone-free interval to the hormone phase demonstrated a pooled SMD of 1.22 (95% CI: 0.81 to 1.62, k=3). Between-group studies comparing HC users to non-users yielded a pooled SMD of -0.57 (95% CI: -1.20 to 0.07, k=3). Stratification by study design also demonstrated significant heterogeneity (p < 0.01): prospective cohort studies (k=4) showed a pooled SMD of 0.86 (95% CI: 0.14 to 1.57), the single cross-sectional study reported an SMD of -0.88 (95% CI: -1.41 to -0.35), and the retrospective cohort study yielded an SMD of -0.94 (95% CI: -1.42 to -0.45). Residual heterogeneity remained substantial following subgroup analyses (I²=94%, τ²=0.98, χ²=67.64, df=4, p<0.01).

Leave-one-out sensitivity analysis indicated that no single study was disproportionately influential. The pooled SMD ranged from 0.05 to 0.53 across iterations, with all confidence intervals crossing zero. Heterogeneity remained substantial (I² = 90.3-93.4%) regardless of which study was excluded. Publication bias was not assessed as the number of studies was <10.

**Figure 3:**
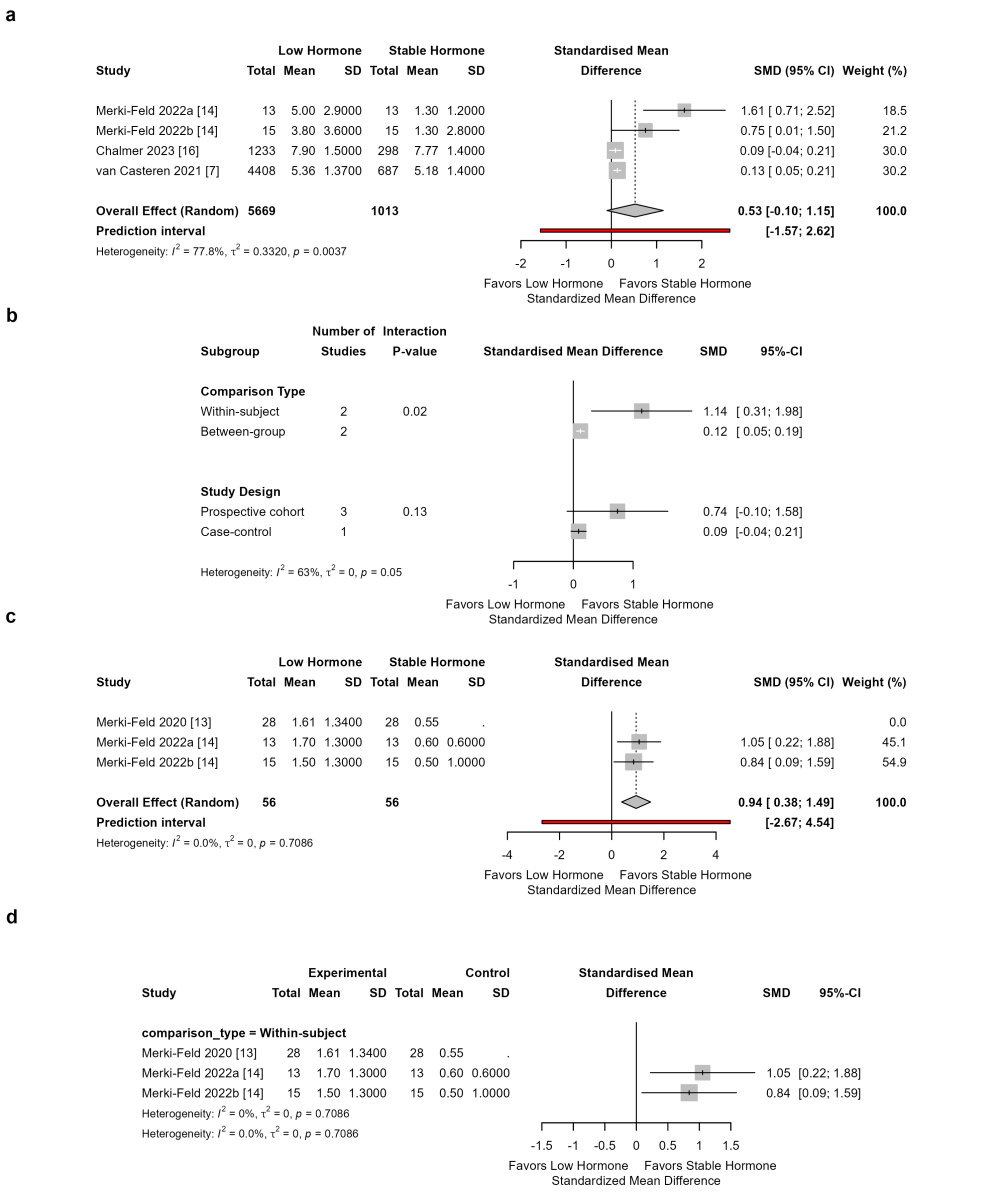
Meta-analysis of migraine severity and acute medication use comparing hormonal contraceptive (HC) users versus non-users. Figure 3. Meta-analysis of migraine severity and acute medication use comparing hormonal contraceptive (HC) users versus non-users. (a) Forest plot for migraine severity [7,14,16]. (b) Subgroup analyses for severity by comparison type and study design. (c) Forest plot for acute medication use [13,14]. (d) Subgroup analysis for medication use by comparison type.

Migraine severity: Four studies comprising 6682 participants were included in the analysis of migraine severity (Figure 3). The pooled SMD was 0.53 (95% CI: -0.10 to 1.15), with substantial heterogeneity (I²=77.8%, τ²=0.33, p=0.0037) and a prediction interval of -1.57 to 2.62. Subgroup analysis by comparison type revealed significant effect modification (p = 0.02): within-subject studies showed higher severity during low hormone states (SMD=1.14, 95% CI: 0.31 to 1.98, k=2), while between-group studies showed a small but statistically significant effect (SMD=0.12, 95% CI: 0.05 to 0.19, k=2). Stratification by study design did not reach statistical significance (p=0.13). Leave-one-out analysis identified Merki-Feld 2022a as an influential study; when excluded, the pooled SMD decreased to 0.12 (95% CI: 0.06 to 0.19) with reduced heterogeneity (I²=35.5%). Exclusion of other individual studies did not meaningfully alter the pooled estimate (SMD range: 0.51-0.74).

Acute medication use: Three within-subject studies (n=112) were pooled for acute medication use. The SMD was 0.94 (95% CI: 0.54 to 1.33), with no heterogeneity (I²=0%, τ²=0, p=0.93) and a prediction interval of 0.08 to 1.80. All three studies were prospective cohorts examining within-subject comparisons, precluding subgroup analysis. Leave-one-out analysis was robust (pooled SMD range 0.90-0.97 across iterations, all confidence intervals excluding zero).

Meta-analysis of associated symptoms (nausea, vomiting, photophobia, phonophobia) was not performed due to the limited number of eligible studies. Only two studies [7, 16] reported these outcomes stratified by HC use. Although Merki-Feld 2022 reported associated symptom prevalence, the comparison was between women whose migraine onset preceded versus followed CHC initiation, representing a fundamentally different research question than HC versus non-HC comparisons. Furthermore, the two available studies differed in their unit of analysis [7, 16].

Discussion

Migraine Burden During the Hormone-Free Interval

We performed a systematic review and meta-analysis to assess the relationship between hormonal contraceptive use and migraine frequency, severity, and acute medication requirements in women with diagnosed migraine. Our synthesis of seven observational studies showed that the association between hormonal contraceptives and migraine outcomes varies considerably depending on the analytical approach used. Within-subject comparisons showed a substantial increase in migraine frequency during the hormone-free interval compared to the hormone phase, whereas between-group comparisons of hormonal contraceptive users and non-users revealed no significant differences. These results represent the first meta-analytic evidence that the hormone-free interval, rather than hormonal contraceptive use per se, is the critical period for migraine burden in women using cyclic combined hormonal contraceptives.

Our pooled analysis found a significant increase in migraine frequency during the hormone-free interval compared to the hormone phase, with within-subject studies yielding a standardized mean difference of 1.22 (95% CI: 0.81-1.62). This finding is consistent with the well-established estrogen withdrawal hypothesis, which holds that falling estrogen levels trigger migraine attacks [5, 9, 20]. The sudden drop in exogenous estrogen during the pill-free week mimics the natural perimenstrual estrogen decline, creating a predictable window of heightened vulnerability [21, 22]. We also found that migraine attacks during this period were more painful and associated with significantly greater acute medication use (pooled SMD: 0.94, 95% CI: 0.54-1.33). Diary-based prospective studies by Merki-Feld and colleagues showed a four- to five-fold increase in migraine risk during the hormone-free interval, with attacks clustered on days three to six after hormone withdrawal [13, 15]. The consistency of this pattern across multiple prospective studies supports the biological plausibility of estrogen withdrawal as a key mechanism underlying menstrual migraine in combined hormonal contraceptive users. As all included studies were observational, these findings establish an association but cannot confirm causation.

Between-Group Comparisons and Clinical Phenotype Insert

In contrast to the within-subject findings, the between-group meta-analysis comparing hormonal contraceptive users to non-users revealed no statistically significant differences in migraine frequency or severity. The large population-based study by Chalmer et al. confirmed this pattern, with women experiencing spontaneous menstrual migraine reporting similar attack frequency, pain intensity, and treatment response as those with hormonal contraceptive-related menstrual migraine [16]. Van Casteren et al. and Verhagen et al. also found comparable migraine attack characteristics between women using hormonal contraception and those with natural menstrual cycles, including similar migraine day counts and medication patterns [7, 17]. However, Mas-de-les-Valls et al. reported that hormonal contraceptive users had a significantly higher risk of headache during ovulation and perimenstrual windows compared to non-users, suggesting that cyclical vulnerability patterns may differ by contraceptive status [18]. These findings suggest that while the overall migraine phenotype appears similar regardless of contraceptive status when comparing different individuals, the timing of attacks may vary.

The apparent discrepancy between within-subject and between-group findings has important methodological implications. Within-subject designs capture intra-individual hormonal fluctuation effects by comparing the same woman during different phases of her contraceptive cycle, thereby controlling for individual-level confounders [14]. Between-group designs, while useful for comparing overall disease burden across populations, may obscure cyclical patterns because they aggregate data from women with varying baseline characteristics and hormonal sensitivities. This fundamental methodological difference likely explains the substantial heterogeneity observed in our overall pooled estimate (I²=91.7%).

Residual heterogeneity remained substantial (I²=94%) even after stratification by comparison type, indicating that additional sources of variability beyond the within-subject versus between-group distinction contribute to between-study differences. These likely include differences in hormonal contraceptive formulations (ethinylestradiol dose, progestin type), outcome ascertainment (prospective diary vs retrospective recall), follow-up duration, and study populations (specialist headache clinics vs population-based samples). The limited number of included studies precluded meta-regression to formally quantify the contribution of individual moderators. Notably, heterogeneity within each subgroup was considerably lower than the overall pooled estimate, suggesting that the within-subject versus between-group distinction captures the predominant source of variability, with the residual overall I² reflecting the large contrast between subgroup effect sizes.

Mechanistic Considerations

Emerging mechanistic evidence provides a biological basis for the observed patterns. The cross-sectional study by Storch et al. [12] found that women with migraine and a natural menstrual cycle had significantly higher pituitary adenylate cyclase-activating polypeptide-38 (PACAP-38) concentrations during menstruation compared to combined oral contraceptive users during the hormone-free interval (2547 vs. 1504 pg/mL, p=0.040). This neuropeptide is increasingly implicated in migraine pathophysiology, with potential roles in trigeminovascular activation and neurogenic inflammation [23-25]. Calcitonin gene-related peptide (CGRP), another key mediator in migraine, interacts with estrogen in a sex-dependent manner and may influence attack susceptibility [26-28]. Raffaelli and colleagues reported that women with episodic migraine and regular menstrual cycles had significantly higher CGRP concentrations during menstruation compared to combined oral contraceptive users [11]. Although direct measurement of CGRP or PACAP-38 was not reported in the included clinical-outcome studies, the observed increase in migraine burden during the hormone-free interval is mechanistically consistent with estrogen-withdrawal-induced upregulation of these neuropeptides. However, these findings should be interpreted cautiously, given the cross-sectional designs and lack of direct correlation with clinical outcomes.

Clinical Implications

These findings have practical clinical implications. The concentration of migraine burden during the hormone-free interval suggests that strategies targeting this period may be particularly beneficial. Extended or continuous hormonal contraceptive regimens that eliminate or shorten the hormone-free interval reduce the frequency of estrogen-withdrawal windows and have been associated with fewer migraine days compared with standard 21/7 regimens [13, 29, 30]. Short-term perimenstrual prophylaxis administered around the hormone-free interval offers an alternative strategy when continuous regimens are not preferred [31].

Beyond migraine burden, the choice of a hormonal contraceptive in women with migraine must also be weighed against cerebrovascular risk. Migraine with aura is an independent risk factor for ischemic stroke, and combined hormonal contraceptives containing ethinylestradiol further increase this risk [32, 33]. Current guidance from the Faculty of Sexual and Reproductive Healthcare (FSRH), the European Headache Federation (EHF), and the American College of Obstetricians and Gynecologists (ACOG) therefore restricts the use of combined hormonal contraceptives in women with migraine with aura and recommends progestin-only or non-hormonal alternatives [34-36]. These guidelines must be read alongside efficacy-related findings such as ours, since regimen choice involves balancing migraine-symptom control against vascular safety.

Limitations

Several limitations should be acknowledged. The predominance of observational designs precludes causal inferences, and the possibility of selection bias cannot be excluded. The cross-sectional nature of some included studies limits assessment of temporal relationships between hormone exposure and migraine outcomes. Heterogeneity in hormonal contraceptive formulations across studies represents another limitation, as different progestin types and estrogen doses may have varying effects on migraine patterns. Residual confounding by contraceptive formulation, patient selection, and baseline migraine characteristics cannot be excluded. The small number of eligible studies (k=7 overall, with three to six studies contributing to individual meta-analyses) limits statistical power for detecting subgroup differences, increases the risk of chance findings, and precludes formal assessment of publication bias. Several individual studies also had small sample sizes (n=28-113), which may have led to imprecise effect estimates. Additionally, most studies enrolled women from headache specialty clinics, potentially limiting generalizability to community populations. The variation in outcome definitions and measurement instruments across studies contributed to statistical heterogeneity.

Future research should prioritize randomized controlled trials comparing different hormonal contraceptive regimens, particularly extended versus cyclic formulations, in women with migraine. Investigation of biomarker dynamics, including PACAP and CGRP, alongside clinical outcomes, may clarify mechanistic pathways linking exogenous hormones, neuropeptide signaling, and migraine susceptibility. Given the emerging evidence for differential efficacy of CGRP-targeting therapies during perimenstrual versus non-perimenstrual periods, trials specifically evaluating these agents for hormone-withdrawal migraine are warranted.

## Conclusions

In conclusion, our systematic review demonstrates that the hormone-free interval represents a critical period of heightened migraine burden in women using cyclic combined hormonal contraceptives, with increased attack frequency, severity, and medication requirements. These findings support clinical strategies targeting the hormone-free interval, including extended regimens and short-term perimenstrual prophylaxis, while highlighting the need for randomized trials to guide evidence-based management.

## Appendices

Detailed search strategy

All database searches were last executed on 26 December 2025

PubMed

(("Contraceptives, Oral, Hormonal"[Mesh] OR "Contraceptives, Oral, Combined"[Mesh] OR "Contraceptive Agents, Female"[Mesh] OR "Progestins"[Mesh] OR "Ethinyl Estradiol"[Mesh] OR "Levonorgestrel"[Mesh] OR "Desogestrel"[Mesh] OR "Intrauterine Devices, Medicated"[Mesh] OR "hormonal contracept*"[tiab] OR "oral contracept*"[tiab] OR "combined oral contracept*"[tiab] OR "combined hormonal contracept*"[tiab] OR COC[tiab] OR CHC[tiab] OR "progestin-only pill*"[tiab] OR "progestogen-only pill*"[tiab] OR POP[tiab] OR "contraceptive patch"[tiab] OR "contraceptive ring"[tiab] OR "vaginal ring"[tiab] OR NuvaRing[tiab] OR "contraceptive implant*"[tiab] OR Nexplanon[tiab] OR "hormonal IUD"[tiab] OR "hormonal intrauterine*"[tiab] OR "levonorgestrel IUD"[tiab] OR Mirena[tiab] OR DMPA[tiab] OR "depot medroxyprogesterone"[tiab] OR ethinylestradiol[tiab] OR "ethinyl estradiol"[tiab] OR etonogestrel[tiab] OR drospirenone[tiab] OR dienogest[tiab] OR norethindrone[tiab] OR norgestimate[tiab] OR gestodene[tiab] OR "hormone-free interval"[tiab] OR "pill-free interval"[tiab]) AND ("Migraine Disorders"[Mesh] OR "Migraine with Aura"[Mesh] OR "Migraine without Aura"[Mesh] OR migraine*[tiab] OR "menstrual migraine"[tiab] OR "menstrually-related migraine"[tiab] OR "menstrually related migraine"[tiab] OR "pure menstrual migraine"[tiab] OR "catamenial migraine"[tiab])) Filters: 2020:2025[dp], English[lang]

Scopus

( TITLE-ABS-KEY ( "hormonal contracept*" OR "oral contracept*" OR "combined oral contracept*" OR "combined hormonal contracept*" OR COC OR CHC OR "progestin-only pill*" OR "progestogen-only pill*" OR POP OR "contraceptive patch" OR "contraceptive ring" OR "vaginal ring" OR NuvaRing OR "contraceptive implant*" OR Nexplanon OR "hormonal IUD" OR "hormonal intrauterine*" OR "levonorgestrel IUD" OR Mirena OR DMPA OR "depot medroxyprogesterone" OR ethinylestradiol OR "ethinyl estradiol" OR etonogestrel OR drospirenone OR dienogest OR norethindrone OR norgestimate OR gestodene OR "hormone-free interval" OR "pill-free interval" ) ) AND ( TITLE-ABS-KEY ( migraine* OR "menstrual migraine" OR "menstrually-related migraine" OR "menstrually related migraine" OR "pure menstrual migraine" OR "catamenial migraine" ) ) AND PUBYEAR > 2019 AND PUBYEAR < 2026 AND ( LIMIT-TO ( LANGUAGE , "English" ) )

Web of Science (Core Collection)

TS=(("hormonal contracept*" OR "oral contracept*" OR "combined oral contracept*" OR "combined hormonal contracept*" OR COC OR CHC OR "progestin-only pill*" OR "progestogen-only pill*" OR POP OR "contraceptive patch" OR "contraceptive ring" OR "vaginal ring" OR NuvaRing OR "contraceptive implant*" OR Nexplanon OR "hormonal IUD" OR "hormonal intrauterine*" OR "levonorgestrel IUD" OR Mirena OR DMPA OR "depot medroxyprogesterone" OR ethinylestradiol OR "ethinyl estradiol" OR etonogestrel OR drospirenone OR dienogest OR norethindrone OR norgestimate OR gestodene OR "hormone-free interval" OR "pill-free interval") AND (migraine* OR "menstrual migraine" OR "menstrually-related migraine" OR "menstrually related migraine" OR "pure menstrual migraine" OR "catamenial migraine")) Timespan: 2020-01-01 to 2025-12-31 Languages: English Document Types: Article, Review (exclude if preferred)

Operational definitions

**Migraine frequency** refers to the number of migraine days or discrete migraine attacks per month, menstrual cycle, or other defined time period, measured quantitatively.

**Migraine severity** refers to pain intensity assessed using validated instruments (e.g., Visual Analog Scale, Numerical Rating Scale) or standardized categorical scales (mild, moderate, severe).

**Hormonal contraceptive exposure** includes current use, past use, or initiation of hormonal contraceptives, with assessment of change in migraine outcomes attributable to the exposure. Acceptable hormonal contraceptive types include combined oral contraceptives (COC), progestin-only pills (POP), contraceptive patch, vaginal ring, subdermal implant, injectable contraceptives, and hormonal intrauterine devices (IUD).

**Established migraine diagnosis** refers to a diagnosis of migraine made according to recognized diagnostic criteria (e.g., ICHD-2, ICHD-3) or by physician/neurologist assessment prior to or at the time of study enrollment.

Data transformation

For studies reporting median and interquartile range (IQR), mean, and standard deviation were estimated using the method described by Wan et al. (SD ≈ IQR/1.35), assuming approximately normal distributions. For studies reporting median and range, the range/4 approximation was applied. Where standard deviations were missing for one comparison group within a study (Merki-Feld et al. 2020), imputation was performed using the SD ratio derived from methodologically similar studies by the same research group (Merki-Feld et al. 2022a, 2022b). Sensitivity analyses were planned to assess the robustness of findings to these imputation assumptions.
